# Associations between home deaths and end-of-life nursing care trajectories for community-dwelling people: a population-based registry study

**DOI:** 10.1186/s12913-019-4536-9

**Published:** 2019-10-15

**Authors:** Camilla Kjellstadli, Ling Han, Heather Allore, Elisabeth Flo, Bettina S. Husebo, Steinar Hunskaar

**Affiliations:** 1 Department of Global Public Health and Primary Care, University of Bergen, PO Box 7804, N-5018 Bergen, Norway; 2 Department of Internal Medicine, Yale University School of Medicine, 300 George St Suite 775, New Haven, CT 06511 USA; 3 Department of Biostatistics, Yale School of Public Health, New Haven, CT USA; 4 Department of Clinical Psychology, University of Bergen, PO box 7804, N-5018 Bergen, Norway; 5 Municipality of Bergen, Bergen, Norway; 6 National Centre for Emergency Primary Health Care, NORCE Norwegian Research Centre, Bergen, Norway

**Keywords:** Home care, Epidemiology, Primary care, Terminal care

## Abstract

**Background:**

Few studies have estimated planned home deaths compared to actual place of death in a general population or the longitudinal course of home nursing services and associations with place of death. We aimed to investigate trajectories of nursing services, potentially planned home deaths regardless of place of death; and associations of place of death with potentially planned home deaths and nursing service trajectories, by analyzing data from the last 90 days of life.

**Methods:**

A retrospective longitudinal study with data from the Norwegian Cause of Death Registry and National registry for statistics on municipal healthcare services included all community-dwelling people who died in Norway 2012–2013 (*n* = 53,396). We used a group-based trajectory model to identify joint trajectories of home nursing (hours per week) and probability of a skilled nursing facility (SNF) stay, each of the 13 weeks leading up to death. An algorithm estimated potentially planned home deaths. We used a multinomial logistic regression model to estimate associations of place of death with potentially planned home deaths, trajectories of home nursing and short-term SNF.

**Results:**

We identified four home nursing service trajectories: no (46.5%), accelerating (7.6%), decreasing (22.1%), and high (23.5%) home nursing; and four trajectories of the probability of a SNF stay: low (69.0%), intermediate (6.7%), escalating (15.9%), and increasing (8.4%) SNF. An estimated 24.0% of all deaths were potentially planned home deaths, of which a third occurred at home. Only high home nursing was associated with increased likelihood of a home death (adjusted relative risk ratio (aRRR) 1.29; CI 1.21–1.38). Following any trajectory with elevated probability of a SNF stay reduced the likelihood of a home death.

**Conclusions:**

We estimated few potentially planned home deaths. Trajectories of home nursing hours and probability of SNF stays indicated possible effective palliative home nursing for some, but also missed opportunities of staying at home longer at the end-of-life. Continuity of care seems to be an important factor in palliative home care and home death.

## Background

Like Japan, Germany, Italy and Portugal, Norway has declining home death rates, with only 13.3% home deaths in 2016 [1–6]. Most people, however, express a wish to receive end-of-life care at home or to die at home [7]. We recently estimated that only about half of the registered home deaths in Norway may have been planned to take place at home [8]. Currently, no studies have estimated the number of potentially planned home deaths in comparison to actual place of death in a general population.

Specialized community-based palliative homecare benefits patients by increasing the likelihood of dying at home [9–11], but is unavailable to most dying people [12]. Specialized palliative care is organized within hospitals and mainly focused on cancer patients [13]. Norway has universal healthcare, and municipalities are required to provide home nursing services and skilled-nursing facility care to its inhabitants. Services are available based on needs, and provided to almost 7% of the population. Home nursing services are free to the patient, while skilled nursing facility (SNF) stays have a deductible based on income. Most SNFs offer some palliative care [13]. Community-dwelling people may experience various patterns of home nursing services and short-term SNF stays before death. Few have investigated the longitudinal course of home nursing services and whether it is associated with place of death [14, 15]. Insight into relationships of these services for community-dwelling patients on place of death may inform policy for end-of-life home-based services.

We aimed to 1) investigate trajectories of nursing services in the last 90 days of life; 2) estimate how many deaths that potentially could have been planned home deaths, regardless of actual place of death; and 3) investigate associations between place of death, potentially planned home deaths and nursing service trajectories, by analyzing data from the last 90 days of life.

## Methods

### Study design and data sources

**Fig. 1:**
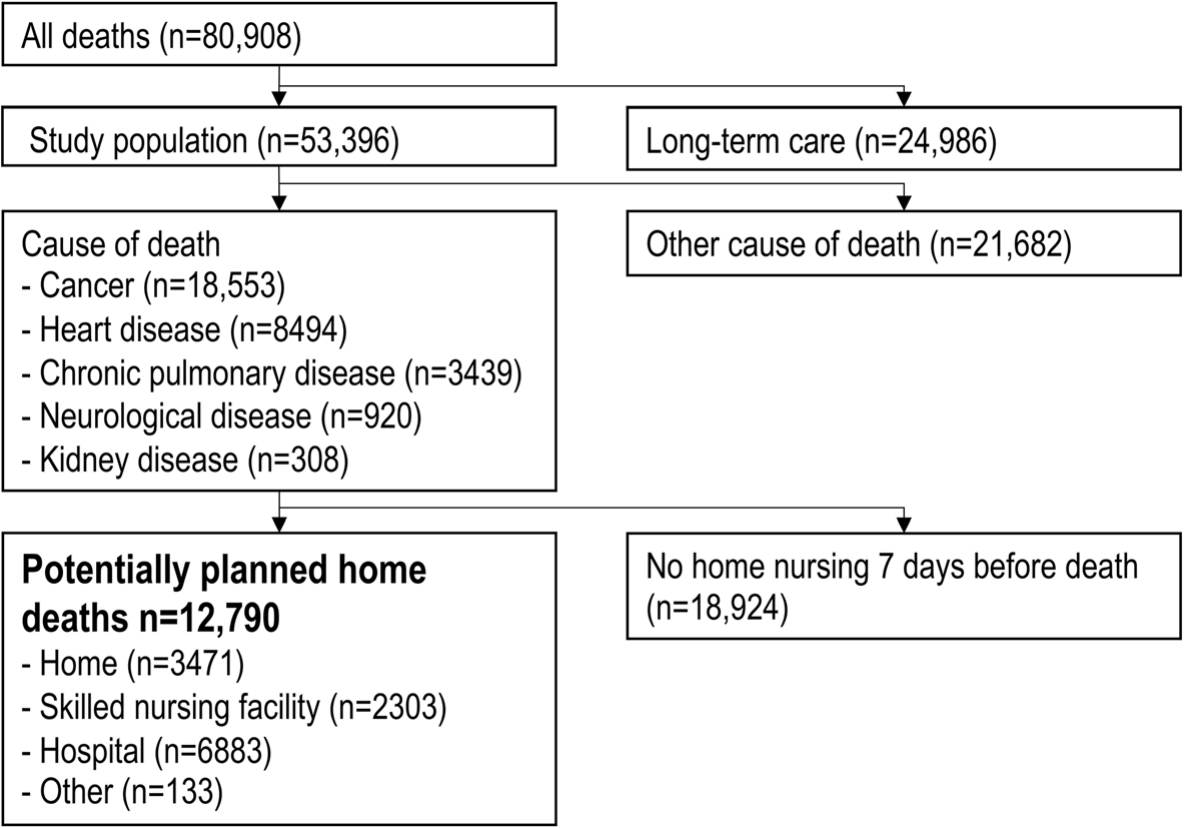
Algorithm to indirectly assess deaths that were potentially planned to occur at home, regardless of actual location of death. Deaths in all locations were assessed (home, hospital, skilled nursing facility, other). Step 1: Cause of death associated with palliative care (y/n). Step 2: Received home nursing services 7 days before death (y/n)

We linked data from the Norwegian Cause of Death Registry (NCoDR) and the National register for statistics on municipal healthcare services (IPLOS) and included all deceased individuals in Norway in 2012–2013 with known place of death and sex (*n* = 80,908) (Fig. 1). We excluded persons in long-term SNFs (*n* = 27,512) to get a study population of community-dwelling people. NCoDR provided information on cause and place of death, age, sex, and municipality centrality. To ensure privacy, people 0–39 years were given fewer details for the cause of death. IPLOS provided information on cohabitation and municipal nursing and care services 0–90 days before death. Information on cohabitation was missing for persons never registered in IPLOS.

### Measurements

#### Home nursing and short-term SNF stays

Home nursing includes specific nursing procedures, such as personal care and daily tasks, drug administration, food preparation and general palliative care. Estimates of the amount of home nursing hours were based on service decisions provided as mean hours per week (hrs/wk) for each of the 13 weeks (0–90 days) leading up to death. People with no home nursing had 0 hrs/wk. and maximum value indicating care 24/7 was 168 hrs/wk. Short-term SNF stays were based on service decisions and coded as occurring or not for each of the 13 weeks leading up to death.

#### Potentially planned home deaths

Based on previous research we developed an algorithm to indirectly estimate deaths that could have been planned to occur at home, regardless of actual location of death (Fig. 1) [8]. A potentially planned home death was considered probable for people with a cause of death most likely to receive palliative care. According to the European Shortlist for Causes of Death, this was ‘Cancer’ (2.), ‘Heart disease’ (7.0/7.1.2/7.2/7.4; excluding acute myocardial infarction), ‘Chronic pulmonary disease’ (8.0/8.3/8.3.1/8.3.2/8.4), ‘Kidney disease’ (12.1) or ‘Neurological disease’ (6.0/6.1/6.3) [16, 17]. Dementia was not included as almost all Norwegians with dementia die in long-term SNFs [8, 18]. Receiving any home nursing 7 days before death was considered a requirement for a planned home death. Thus, in our algorithm, a potentially planned home death required a “yes” to both the following steps of inquiry: Step 1: Was the person’s cause of death associated with palliative care? (y/n), Step 2: Did the person receive home nursing 7 days before death? (y/n). The remaining deaths were categorized as unplanned to take place at home, hereafter ‘unplanned’. To test how sensitive the estimated number of potentially planned home deaths were to changes in the home nursing criterium, we evaluated the effects of replacing receipt of home nursing within day seven with receipt of home nursing 14 days before death. As circumstances may have led to a transition to another location before death, we used the above algorithm to assess deaths in all locations; home, SNF, hospital and other.

#### Covariates

Cause of death was divided into categories based on diagnoses used to define potentially planned home deaths: ‘Cancer’; ‘Heart; ‘Pulmonary’; ‘Kidney’; ‘Neurological’. All other causes were labeled ‘Other’. We defined seven age-groups; 0–39 years, 10-year intervals up to 89 years, and ≥ 90 years. Municipality centrality was defined as a municipality’s geographic location in relation to a center with important central functions, where 0 is least central and 3 most central [19].

### Statistical analyses

Decedent characteristics were presented as frequency and percentages with differences within place of death tested using Chi-square tests.

We used a group-based, dual-trajectory model to identify parallel trajectories of home nursing and short-term SNF stays in the last 13 weeks of life by means of a Stata Traj plugin [20, 21]. This is a semiparametric finite mixture model for longitudinal data using a maximum likelihood method [21]. Hours of home nursing trajectories were modeled using a censored normal distribution after a log transformation (log_10_(home nursing hrs/wk. + 0.1)) to normalize. We modeled probability of a SNF stay each week with a Bernoulli distribution. We modeled each outcome separately, then jointly. Model selection was performed by adding one trajectory at a time followed by varying higher-order growth terms until an optimal fit was achieved based on the Bayesian Information Criterion, average posterior probability of assignment (PPA) (≥0.9 considered excellent fit), odds of correct classification, the proportion with PPA < 0.7 (indicated poor fit), and differences between predicted and observed group proportions [21]. Group size of 5% was considered a minimum.

Next, we used a multinomial logistic regression to estimate associations of place of death with potentially planned home deaths, trajectories of home nursing and short-term SNF. Adjusted relative risk ratio (aRRR) and their 95% CI was estimated after, adjusting for sex, age and municipality centrality as potential confounding factors. Living with others was assessed as a possible confounder in the population with information on cohabitation (registered in IPLOS, *n* = 35,600), without any indication of this being the case. All analyses were conducted with Stata version 15 (Stata Corp, College Station, TX). Two-sided *p*-values < 0.05 were considered statistically significant.

## Results

### Characteristics of the population

In our population of community-dwelling people, 54.1% were men and 85.8% were ≥ 60 years (Table 1). The most common causes of death were cancer (34.8%) and heart disease (15.9%). Almost half died in hospitals, nearly a third in SNFs, and another fifth at home. A higher proportion of men died at home and in hospitals, while women died more frequently in SNF (Table 1). As expected, people ≥80 years had a higher proportion of SNF deaths. Nearly a fifth of home deaths occurred in people < 60 years. While over half of SNF deaths were from cancer, they constituted only a fifth of home deaths. Conversely, deaths from heart disease were more common at home. Ninety days before death 4.9% had a short-term SNF stay, 42.4% received home nursing services, 8.6% received other municipal services, and 44.1% received no municipal services.

**Table 1:** Characteristics of 53,396 home-dwelling people who died in Norway 2012–2013 by place of death

	Home		Nursing home		Hospital		Other^a^	
	n	%	n	%	n	%	n	%
Overall population	11,867	22.2	14,895	27.9	24,241	45.4	2393	4.5
Sex								
Female	4985	42.0	7827	52.6	11,136	45.9	566	23.7
Male	6882	58.0	7068	47.5	13,105	54.1	1827	76.4
Age (years)								
0–39	548	4.6	63	0.4	789	3.3	426	17.8
40–49	549	4.6	165	1.1	719	3.0	263	11.0
50–59	1243	10.5	604	4.1	1854	7.7	389	16.3
60–69	2372	20.0	1860	12.5	4395	18.1	561	23.4
70–79	2505	21.1	3242	21.8	5772	23.8	378	15.8
80–89	3171	26.7	5855	39.3	7827	32.3	314	13.1
90+	1479	12.5	3106	20.9	2885	11.9	62	2.6
Cause of death								
Heart	2456	20.7	1933	13.0	3704	15.3	401	16.8
Cancer	2624	22.1	7629	51.2	8198	33.8	102	4.3
Pulmonary	738	6.2	783	5.3	1874	7.7	44	1.8
Neurological	234	2.0	242	1.6	428	1.8	16	0.7
Kidney	51	0.4	101	0.7	152	0.6	4	0.2
Other	5764	48.6	4207	28.2	9885	40.8	1826	76.3
Household^b^								
Cohabiting	3067	25.8	6390	42.9	7125	29.4	214	8.9
Living alone	3920	33.0	6906	46.4	7741	31.9	314	13.1
Potentially planned home death								
Yes	3471	29.3	2303	15.5	6883	28.4	133	5.6
No	8396	70.8	12,592	84.5	17,358	71.6	2260	94.4
Home nursing trajectory								
No	6055	51.0	4921	33.0	11,846	48.9	2036	85.1
Accelerating	872	7.4	1144	7.7	2118	8.7	34	1.4
Decreasing	1536	12.9	5653	38.0	4413	18.2	159	6.6
High	3404	28.7	3177	21.3	5864	24.2	164	6.9
SNF trajectory								
Low	10,797	91.0	4204	28.2	19,635	81.0	2320	97.0
Increasing	174	1.5	3462	23.2	901	3.7	20	0.8
Intermediate	601	5.1	1082	7.3	1824	7.5	30	1.3
Escalating	295	2.5	6147	41.3	1881	7.8	23	1.0

Note. Pearson chi-square test comparing place of death: *p* < 0.001 for all categories

^a^Other place of death includes abroad, under transportation to hospital, other specified

^b^17,719 missing household

### Joint trajectories of home nursing services and probability of SNF stays

**Fig. 2:**
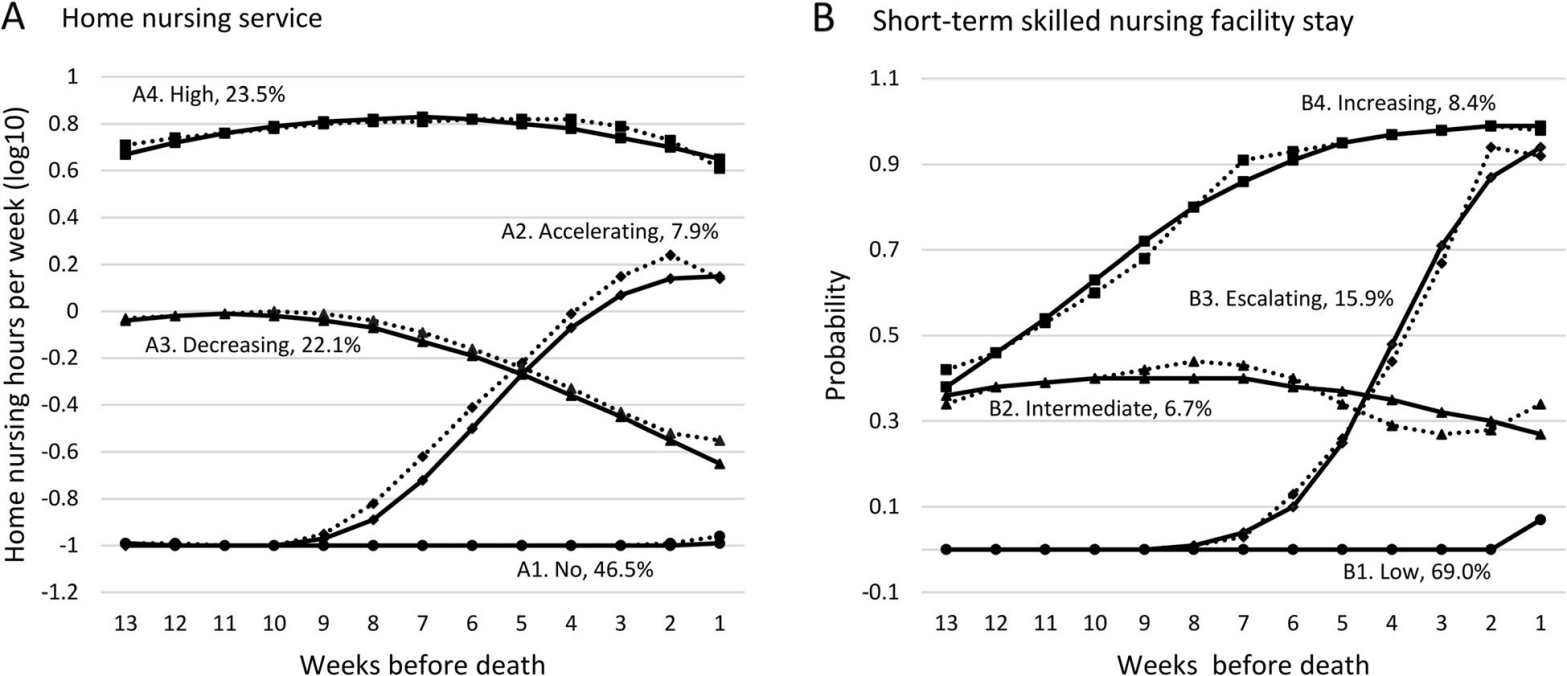
Home nursing service trajectories (**a**) jointly modelled with short-term skilled nursing facility trajectories (**b)** in the last 13 weeks of life. Solid lines represent predicted trajectories, dashed lines indicate observed trajectories. Percentage of population for each trajectory are shown. Home nursing service trajectories were modeled using a censored normal distribution after log transformation. A Bernoulli distribution was used to model probability of a skilled nursing facility stay each week. In total, 97.1% persons had a probability of assigned trajectory ≥ 0.70

We identified four trajectories of home nursing (hrs/wk) and four trajectories of the probability of being in a SNF each week. The model, with quadratic growth terms, was judged to provide an excellent fit to the data, with PPA ≥0.94 for all trajectories, and clinically interpretable. The four trajectories for home nursing services are shown in Fig. 2 (A1-A4):

- A1. The largest group of decedents (46.5%) followed a trajectory of no home nursing services, hereafter called “no home nursing”.
- A2. 7.9% had accelerating home nursing services starting 9 weeks before death, reaching a median of 1.7 hrs/wk. (interquartile range (IQR) 5.8), hereafter called “accelerating home nursing”.
- A3. 22.1% had decreasing home nursing services starting at a median of 1.0 hrs/wk. (IQR 2.1), hereafter called “decreasing home nursing”.
- A4. 23.5% maintained a high level of home nursing services with a median of 6.8 hrs/wk. (IQR 9.2) 5 weeks before death, hereafter called “high home nursing”.

The four trajectories for short-term SNF stays are shown in Fig. 2 (B1-B4):

- B1. 69.0% had a consistently low probability of SNF, hereafter called “low SNF”.
- B2. 6.7% had an intermediate probability of SNF, hereafter called “intermediate SNF”.
- B3. 15.9% had an initial low probability of SNF escalating from 7 weeks before death, hereafter called “escalating SNF”.
- B4. 8.4% had a trajectory with increasing probability of SNF, hereafter called “increasing SNF”.

### Potentially planned home deaths

We estimated that 12,790 (24.0%) deaths were potentially planned to take place at home (Fig. 1). Receiving home nursing 14 days instead of 7 days before death, yielded marginally more (13,603; 25.5%) potentially planned home deaths, resulting in a higher proportion of SNF deaths. Actual place of death for the 12,790 potentially planned home deaths was 27.1% home, 18.0% SNF, 53.8% hospital and 1.0% other locations. In total, only 6.5% of all deaths were potentially planned to take place at home and occurred at home. This corresponds to 15.8% potentially planned home deaths in the entire deceased population in the same period, with 4.3% of all deaths being potentially planned home deaths that occurred at home.

### Comparing potentially planned home deaths and nursing care trajectories

**Fig. 3:**
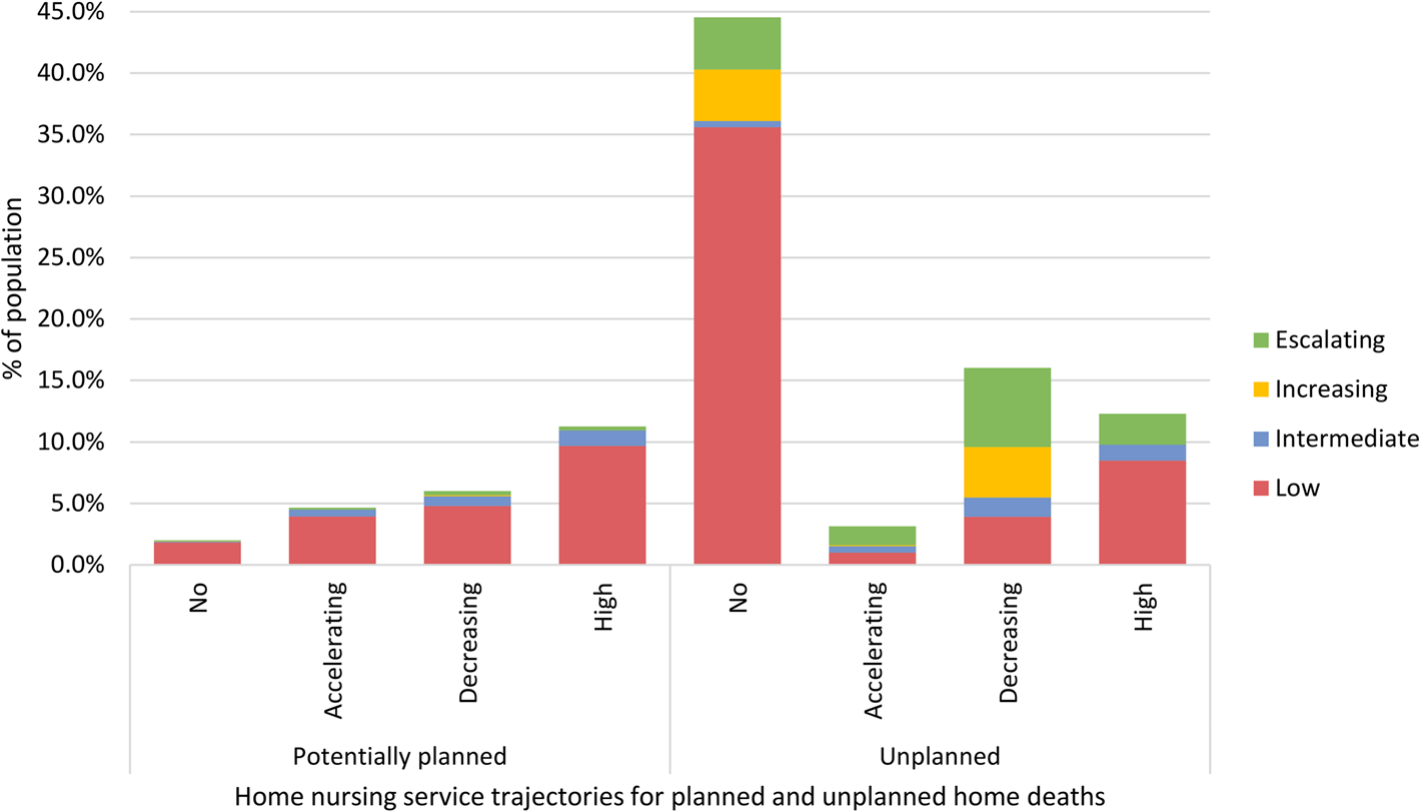
Joint probability of being a member of a specific home nursing service trajectory and a specific short-term skilled nursing facility trajectory for potentially planned and unplanned home deaths. The probabilities sum up to 100%

Nearly half of people with conditions that predicted a potentially planned home death had high home nursing services (11.3%) (Fig. 3). An additional 4.7% of the population had potentially planned home deaths and accelerating home nursing. Almost all patients with potentially planned home deaths had a low probability of going to a nursing home, regardless of which home nursing trajectory they followed. Somewhat unexpectedly, this included those with decreasing home nursing. For people with unplanned home deaths, 4.2 and 4.3% had no home nursing and followed the increasing or escalating SNF trajectories, respectively. In general, people with unplanned home deaths had a larger proportion of people who followed trajectories with increased probability of having a short-term SNF stay towards the end-of-life.

### Associations between place of death, potentially planned home deaths and home nursing service trajectories

We found no significant association between death at home versus hospital or SNF versus hospital and potentially planned home deaths after adjusting for other factors (Table 2).

**Table 2:** Adjusted relative risk ratios (aRRR) for dying at home, skilled-nursing facility or other location compared to hospital and their associations with potentially planned home deaths, home nursing trajectories and skilled nursing facility trajectories

	Home versus Hospital			SNF versus Hospital			Other^a^ versus Hospital		
	aRRR	CI	p	aRRR	CI	p	aRRR	CI	p
Potentially planned home death (ref. unplanned)	0.94	0.89–1.00	0.066	0.96	0.90–1.03	0.264	0.28	0.23–0.34	< 0.001
Home nursing trajectory (ref. no)									
Accelerating	0.93	0.85–1.03	0.168	1.23	1.11–1.36	< 0.001	0.30	0.21–0.43	< 0.001
Decreasing	0.90	0.83–0.97	0.004	1.43	1.34–1.54	< 0.001	0.66	0.55–0.79	< 0.001
High	1.29	1.21–1.38	< 0.001	1.58	1.47–1.70	< 0.001	0.51	0.43–0.61	< 0.001
SNF trajectory (ref. low)									
Increasing	0.40	0.34–0.47	< 0.001	17.93	16.43–19.56	< 0.001	0.19	0.12–0.30	< 0.001
Intermediate	0.65	0.59–0.72	< 0.001	2.27	2.08–2.47	< 0.001	0.37	0.26–0.54	< 0.001
Escalating	0.32	0.28–0.36	< 0.001	14.14	13.21–15.14	< 0.001	0.14	0.09–0.22	< 0.001
Female (ref. male)	0.85	0.81–0.89	< 0.001	1.13	1.07–1.19	< 0.001	0.49	0.44–0.54	< 0.001
Age (years) (ref. 80–89)									
0–39	1.63	1.45–1.84	< 0.001	0.20	0.15–0.27	< 0.001	7.84	6.61–9.31	< 0.001
40–49	1.80	1.59–2.03	< 0.001	0.57	0.46–0.69	< 0.001	6.22	5.15–7.51	< 0.001
50–59	1.59	1.46–1.83	< 0.001	0.71	0.63–0.80	< 0.001	3.75	3.19–4.41	< 0.001
60–69	1.30	1.22–1.39	< 0.001	0.82	0.75–0.89	< 0.001	2.38	2.04–2.76	< 0.001
70–79	1.06	1.00–1.13	0.060	0.91	0.85–0.97	0.005	1.36	1.16–1.59	< 0.001
90+	1.20	1.11–1.30	< 0.001	1.55	1.44–1.67	< 0.001	0.66	0.49–0.87	0.003
Municipality centrality^b^ (ref. central)									
Least central	1.24	1.16–1.34	< 0.001	1.56	1.45–1.69	< 0.001	1.53	1.34–1.75	< 0.001
Less central	1.06	0.97–1.16	0.205	1.12	1.02–1.24	0.023	1.28	1.09–1.51	0.003
Somewhat central	1.13	1.06–1.20	< 0.001	1.21	1.13–1.29	< 0.001	0.99	0.88–1.12	0.868

Note. Multinomial logistic regression with place of death as dependent variable. Number of observations 53,177

*Abbreviations*: *SNF* skilled nursing facility

^a^Other place of death includes abroad, under transportation to hospital, other specified

^b^Classification based on geographical distance to center with higher functions

Only people following the high home nursing trajectory had increased likelihood of dying at home compared to hospital (aRRR 1.29, CI 1.21–1.38) (Table 2). Decreasing home nursing was associated with reduced likelihood of home death (aRRR 0.90, CI 0.83–0.97), while no significant association was found for accelerating home nursing. People following trajectories of high (aRRR 1.58, CI 1.47–1.70), decreasing (aRRR 1.43, CI 1.34–1.54) and accelerating home nursing (aRRR 1.23, CI 1.11–1.36) were all more likely to die in a SNF than hospital. Increasing SNF, escalating SNF and intermediate SNF were all associated with reduced likelihood of a home death and higher likelihood of dying in a SNF compared to hospital. In general, younger age groups were associated with increased likelihood of dying at home and less likelihood of SNF deaths compared to hospital. Those aged ≥90 years were more likely to die both at home and in SNFs than in hospitals. People living in the least central municipalities had the highest likelihood of dying both at home (aRRR 1.24, CI 1.16–1.34) and in SNFs (aRRR 1.56, CI 1.45–1.69), compared to hospitals.

## Discussion

We identified four home nursing service trajectories and four short-term SNF trajectories in the last 3 months of life in this community-dwelling population. An estimated 24.0% were potentially planned home deaths, of which a third occurred at home. Half of people with potentially planned home deaths followed the high home nursing trajectory. Only high home nursing was associated with increased likelihood of dying at home. Following any trajectory with elevated probability of a SNF stay reduced the likelihood of a home death. We believe we are the first to use trajectory modeling to investigate patterns of care for home nursing simultaneously with short-term SNF stays in the last months of life and to calculate associations with place of death.

Strengths of our study are the national coverage and registry-based data. We had access to large numbers of deaths providing higher power, using state-of-the-art modelling and had an excellent fit. Universal healthcare with access to services for all inhabitants in Norway requiring such services, increases validity of our findings. Limitations include lack of information on hospital admissions, date of admission for hospital deaths, and contacts with family physicians or specialized palliative care services. As no registry-based information source was available, we estimated potentially planned home deaths. While receiving home nursing services seven or 14 days before death is a narrow definition, this was considered the latest initiation compatible with building relationships and providing palliative care at home. Almost all people with potentially planned home deaths started home nursing at an earlier time. However, we cannot rule out that some, especially younger people, may have died at home with support from family caregivers and possibly hospital-based specialized palliative care. We could not investigate this further, as information on cohabitation was only available for those who received municipal care. Additionally, we cannot exclude planned home deaths for other diagnoses than those included in our definition. The current algorithm led to 3471 (4.3% of all) home deaths being classified as potentially planned compared with 5089 (6.3%) in our previous publication, because of a refinement of the inclusion criteria [8]. We consider the 24.0% potentially planned home deaths a valid estimate because palliative care is mostly offered to cancer patients and planned home deaths are unlikely without home nursing [17, 22].

People with potentially planned home deaths for the most part had a low probability of having a short-term SNF stay and half received high hours of home nursing. Home nursing service utilization indicates that time at home and possibly home death was prioritized. People receiving high home nursing was also the only group with significantly higher likelihood of home death. A plausible explanation is that people following this trajectory had high care needs over a longer period, received home nursing from familiar caregivers and felt secure staying at home. The evidence from previous studies are conflicting on home nursing and associations with days spent at home [23, 24], and timing of palliative care [25, 26].

Our findings imply that continuity of services is an important factor to stay longer at home and die at home. This is further supported by that we did not find any significant association between accelerating home nursing and home death, although home time seems to have been prioritized also here. Accelerating home nursing started closer to death, never reached the number of hours provided to people receiving high home nursing; and may in the end have been too little, too late to die at home. To have continuity and timely start-up of services, the patient and family’s preferences of place of care and death must be known to healthcare providers. This can be achieved through advance care planning, which has been shown to both increase chances of dying at home and improve quality of care [27, 28].

Cancer patients constituted the largest group in all trajectories receiving home nursing. They also more commonly have advance care planning [27]. This may be attributed to cancer having a terminal phase that is easier to predict [29]. Two-thirds of patients receiving accelerating home nursing services died from cancer and fits well with a response to a well-defined trajectory of rapid functional decline at the end-of-life; mostly attributed to cancer patients [29]. Yet, another cohort-study found that most people did not have a distinct trajectory based on cause of death [30]. There was, however, agreement on substantial functional decline in the last months of life regardless of diagnosis [29, 30]. So if most people with a non-sudden death have rapid functional decline approaching death [29, 30], our findings indicate many missed opportunities to identify and provide palliative homecare to enable people to stay longer at home; especially non-cancer patients.

Home death is not feasible for all dying persons, and for these, transitions to SNF or hospital may be appropriate. To illustrate, people who received high home nursing hours and had escalating probability of a SNF stay most likely represent high care needs over time where declining function, lack of symptom control, high caregiver burden or living alone may have led to a necessary transition. On the other hand, 22% of decedents followed a trajectory of decreasing home nursing services. Of these, 50% were already in a SNF before the last week of life and hence not considered potentially planned home deaths. Another 40% had a low joint probability of a SNF stay. More intensive home nursing services may represent an alternative to SNF or hospital admission at the end-of-life. A majority never received home nursing services and had low probability of SNF stays. Some represent sudden or unexpected deaths, and some younger patients were probably cared for by family caregivers. Still, it is likely that a significantly larger proportion could have benefited from receiving palliative home nursing at an earlier stage [31].

With increasing demand for palliative care regardless of diagnosis, specialized palliative care cannot alone meet the needs of patients and families [32]. A recent Swedish study found that a majority of quality indicators for end-of-life care in the last week of life were better for patients dying in community-based settings in regions with less developed palliative care compared to fully developed palliative care [33]. General palliative care should be provided by all relevant healthcare personnel, while specialist palliative care should manage more complex cases [32]. Together with an involved family physician, home nursing services could be a viable alternative for providing general palliative care to people according to their wishes, regardless of diagnosis [34]. For this to work, we must also address inadequate policies and guidelines, gaps in continuity and coordination of care and increase the knowledge and skills in palliative end-of-life care for all health personnel [34–36].

## Conclusions

Our estimates show a low number of potentially planned home deaths in Norway. Trajectories of home nursing hours and probability of SNF stays indicated possible effective palliative home nursing for some, but also missed opportunities of staying at home longer at the end-of-life. Continuity of care seems to be an important factor in providing home nursing and dying at home. Transitions from home need further research to ascertain if current policies maximize time spent at home and increase the likelihood of home deaths. Future studies should also investigate how family physicians follow up patients at the end-of-life and whether they can contribute to an increased number of planned home deaths.

## Data Availability

The data that support the findings of this study are available from NCoDR and IPLOS but restrictions apply to the availability of these data, which were used under license for the current study, and so are not publicly available. Data are however available from the authors upon reasonable request and with permission of NCoDR and IPLOS.

## Abbreviations

aRRR: Adjusted relative risk ratio
Hrs/wk.: Hours per week
IPLOS: National register for statistics on municipal healthcare services
IQR: Interquartile range
NCoDR: Norwegian Cause of Death Registry
PPA: Average posterior probability of assignment
SNF: Skilled nursing facility
